# Entropy Application for Forecasting

**DOI:** 10.3390/e22060604

**Published:** 2020-05-29

**Authors:** Ana Jesús López-Menéndez, Rigoberto Pérez-Suárez

**Affiliations:** Department of Applied Economics, University of Oviedo, Campus del Cristo s/n, 33006 Oviedo, Asturias, Spain; rigo@uniovi.es

**Keywords:** information theory, uncertainty, forecasting methods, forecasting evaluation, accuracy, M-competition, combined forecasts, scenarios

The information theory developed by Shannon [1] defines the entropy for any probabilistic system as a measure of the related uncertainty. This measure, inspired by the entropy defined in thermodynamics by Boltzmann [2], provides a link between uncertainty and probability and opens a wide variety of applications in different fields.

The basic idea of information theory is that the informational content of a message depends on the degree to which it is surprising: if an event is very likely to occur, there is no surprise when this event happens as expected; on the contrary, it is much more informative to know that an unlikely event has taken place.

In this context, entropy can be understood as a measure of unpredictability and therefore it is not surprising that entropy and information theory can be of great help in a broad range of problems related to forecasting, as shown by Theil [3,4].

The contributions to this Special Issue “Entropy Application for Forecasting” show the enormous potential of entropy and information theory in forecasting, including both theoretical developments and empirical applications.

The contents cover a great diversity of topics, such as the aggregation and combination of individual forecasts [5,6], the comparison of forecasting performances [7,8], the analysis of forecasting uncertainty [9], robustness [10] and inconsistency [11], and the proposal of new forecasting approaches [12,13,14].

A great diversity is also observed in the methods, since the contributions encompass a wide variety of time series techniques (ARIMA, VAR, State Space Models, etc.) as well as econometric methods and machine learning algorithms.

Furthermore, the empiric contents are also diverse including both simulated experiments and real-world applications. More specifically, the contributions provide empirical evidence that refer to the economic growth and gross domestic product (GDP) [5,9], the M4 competition dataset [8], the confidence and industrial trend surveys [9], and some stock exchange composite indices (Taiwan, Shanghai, Hong-Kong) [11], as well as other real data from a Portuguese retailer [7] and a Chinese grid company [12].

In summary, this Special Issue provides an engaging insight into entropy applications for forecasting, offering an interesting overview of the current situation and suggesting possibilities for further research in this field.
